# Highly diluted medication reduces parasitemia and improves experimental infection evolution by *Trypanosoma cruzi*

**DOI:** 10.1186/1756-0500-5-352

**Published:** 2012-07-11

**Authors:** Denise Lessa Aleixo, Fabiana Nabarro Ferraz, Érika Cristina Ferreira, Marta de Lana, Mônica Lúcia Gomes, Benício Alves de Abreu Filho, Silvana Marques de Araújo

**Affiliations:** 1 Laboratory of Parasitology, Universidade Estadual de Maringá, Maringá, PR, Brazil; 2 Laboratory of Parasitology, Universidade Federal de Ouro Preto, Ouro Preto, MG, Brazil; 3 Laboratory of Microbiology, Universidade Estadual de Maringá, Maringá, PR, Brazil

**Keywords:** Biotherapy, Mice infection, Parasitological evaluation, Clinical evaluation, Prepatent period

## Abstract

**Background:**

There is no published information about the use of different protocols to administer a highly diluted medication.

Evaluate the effect of different protocols for treatment with biotherapic *T. cruzi* 17 dH (BIOT_*Tc*17dH_) on clinical/parasitological evolution of mice infected with *T. cruzi-Y* strain.

**Methods:**

A blind, randomized controlled trial was performed twice, using 60 28-day-old male Swiss mice infected with *T. cruzi-Y* strain, in five treatment groups: CI *-* treated with a 7% ethanol-water solution, diluted in water (10 μL/mL) *ad libitum*; *BIOT*_*PI*_ - treated with BIOT_*Tc*17dH_ in water (10 μL/mL) *ad libitum* during a period that started on the day of infection; *BIOT*_*4DI*_ - treated with BIOT_*Tc*17dH_ in water (10 μL/mL) *ad libitum* beginning on the 4^th^ day of infection; *BIOT*_*4-5–6*_ - treated with BIOT_*Tc*17dH_ by gavage (0.2 mL/ animal/day) on the 4^th^, 5^th^ and 6^th^ days after infection; *BIOT*_*7-8–9*_ - treated with BIOT_*Tc*17dH_ by gavage (0.2 mL/ animal/day) on the 7^th^, 8^th^ and 9^th^ days after infection. We evaluated: parasitemia; total parasitemia (P_total_); maximum peak of parasites; prepatent period (PPP) - time from infection to detection of the parasite in blood; patent period (PP) - period when the parasitemia can be detected in blood; clinical aspects; and mortality.

**Results:**

Parasitological parameters in the BIOT_PI_ and mainly in the BIOT_4PI_ group showed better evolution of the infection compared to the control group (CI), with lower P_total_, lower maximum peak of parasites, higher PPP, lower PP and longer survival times. These animals showed stable body temperature and higher weight gain and water consumption, with more animals having normal-appearing fur for longer periods. In contrast, groups BIOT_4-5–6_ and BIOT_7-8–9_ showed worse evolution of the infection compared to the control group, considering both parasitological and clinical parameters. The correlation analysis combined with the other data from this study indicated that the prepatent period is the best parameter to evaluate the effect of a medication in this model.

**Conclusions:**

The BIOT_4DI_ group showed the best clinical and parasitological evolution, with lower parasitemia and a trend toward lower mortality and a longer survival period. The prepatent period was the best parameter to evaluate the effect of a medication in this model.

## Background

The use of highly diluted medications in the treatment of parasitic diseases has been investigated, but the available information does not allow us to demonstrate details of their effects. One of the hypotheses regarding the therapeutic effect of these drugs is that they modulate the immune system, and understanding the dynamics of this effect depends on the recognition of the information systems involved [1,2]. Bellavite *et al.* (2007) [3] suggested that homeopathic medicine can regulate inflammatory and immunopathological processes as well as the neuroendocrine network and peripheral receptors. Specifically in mice infected with *T. cruzi*, one study reported an increase in the apoptosis rate and a decrease in the secretion of TGF-β measured in serum collected from the group treated with biotherapic medication, compared with the control group [4].

Although several studies have attempted to explain the clinical results of highly diluted medications, few trials have evaluated the effect of different treatment schemes with these drugs on living organisms [5-7].

Biotherapics are highly diluted medications prepared according to homeopathic techniques [8] from biological products, including the etiological agent itself [9]. Although experience in clinical practice using biotherapics cannot be ignored, academic research on biotherapics has been questioned [10].

Chagas disease, caused by the protozoan *Trypanosoma cruzi*, has been studied for more than a century. However, no effective drug for etiological treatment has been discovered so far [11].

*T. cruziY* is considered a reference strain [12]. Extensively studied in experimental murine models, it shows well-defined characteristics such as high parasitemia, peak of parasites by the 12^th^ day of infection, death of all untreated infected animals, and partial resistance to drugs classically used in the treatment of human Chagas disease. Therefore, the murine model is an excellent experimental model for the evaluation of drug interventions [13].

According to the literature, morbidity is related to the presence of the parasite [14,15], and pathogenesis increase is the result of a disruption of the host-parasite relationship [16]. Therefore, using biotherapics as an intervention in murine infection with *T. cruzi* is a possible means to understand the effect of these highly diluted medications and to find new candidates to treat Chagas disease.

The present study evaluated *in vivo* the effect of different treatment schemes using the biotherapic *Trypanosoma cruzi* 17dH (BIOT_*Tc*17dH_) in an experimental infection by this protozoan.

## Results and discussion

As far as we are aware, this is the first study to compare different treatment schemes using a biotherapic *T. cruzi* in murine infection by the protozoan. Evaluation of parasitological and clinical parameters showed that the different treatment schemes using this medicine produced different profiles in the evolution of the infection of the Y strain of *T. cruzi* in Swiss mice.

### Parasitological parameters

The mean and standard deviation of the parasitological parameters for each group are shown in Tables 1 and 2. The evolution of parasitemia in groups of animals treated with biotherapics diluted in water *ad libitum* for a long period (BIOT_PI_ and BIOT_4PI_) showed better evolution of the infection compared to the control group (CI).

**Table 1:** **Parasitological parameters of animals subjected to different treatment schemes using biotherapic (BIOT**_***Tc*17dH**_**)**

**GROUP**	**AUC (x10**^**5**^**)**	**P**_**total**_**(x10**^**6**^**)**	**P**_**max**_**(x10**^**6**^**)**	**DAY OFP**_**max**_	**P**_**8thDI**_**(x10**^**6**^**)**	**2**^**nd**^** P**_**max**_**(x10**^**4**^**)**	**DAY OF 2**^**nd**^** PEAK**	**2**^**nd**^** PEAK n/N (%)**
CI	10.9±8.20	12.5±8.98	5.02±7.07	10.76±3.15	2.82±3.12	11.0±10.2	14.03±2.63	8/12 (66%)
BIOT_4-5-6_	9.23±3.26	9.33±3.25	5.82±2.14**	8.25±0.67**	5.11±2.62	2.10±17.4	12.0±2.0*	4/12 (33%)
BIOT_7-8-9_	8.78±2.34	9.32±2.48	4.05±2.42	8.64±0.71**	3.96±2.51	17.4±0	14.25±1.02	8/12 (66%)
BIOT_PI_	5.95±4.55**	6.63±5.02**	2.25±1.65**	15.46±23.06	1.62±1.69	11.1±13.6	13.67±0.63*	6/12 (50%)
BIOT_4PI_	4.28±2.89**	4.63±3.19**	1.45±1.00**	10.27±2.03	1.01±0.96	7.09±10.2	12.8±0.68*	6/12 (50%)

Statistical significance (p < 0.05* and p < 0.01**) compared to control group: CI: control group – infected animals treated with 7% ethanol-water solution diluted in water (10μL/mL) offered *ad libitum*; BIOT_PI_: infected animals treated with biotherapic BIOT_*Tc*17dH_ diluted in water (10μL/mL) offered *ad libitum* from the day of the infection until the death of the animals; BIOT_4PI_: infected animals treated with biotherapic BIOT_*Tc* 17dH_ diluted in water (10μL/mL) offered *ad libitum* from the 4^th^ day of the infection until the death of the animals; BIOT_4-5–6_: infected animals treated with biotherapic BIOT_*TC* 17dH_ by gavage on the 4^th^, 5^th^ and 6^th^ day of the infection; BIOT_7-8–9_: infected animals treated with biotherapic BIOT_*TC* 17dH_ by gavage on the 7^th^, 8^th^ and 9^th^ day of the infection. AUC = area under the curve (parasites/ mL); P_total_ = total parasitemia (parasites/ mL); P_max_ = maximum peak of parasites (parasites/ mL); Day of P_max_ = Day of maximum peak of parasites; P_8thDI_ = number of tripomastigotes on 8^th^ day of infection (parasites/ mL); 2^nd^ P_max_ = number of tripomastigotes in the 2^nd^ peak of parasites (parasites/ mL); Day of 2^nd^ P_max_ = Day of 2^nd^ peak of parasites; 2^nd^PEAK n/N = number of animals that showed 2^nd^ peak of parasites (n)/ total number of infected animals (N) x 100.

**Table 2:** **Prepatent period (PPP), patent period (PP), survival and percent mortality of animals subjected to different treatment schemes using biotherapic (BIOT**_***Tc*17dH**_**)**

**GROUP**	**PPP (days)**	**PP (days)**	**SURVIVAL (days)**	**DEATH n/N (%)**
CI	6.02±1.85	9.63±2.74	15.33±3.39	12/12 (100%)
BIOT_4-5-6_	3.86±1.36*	7.43±1.99*	12.14±1.25*	12/12 (100%)
BIOT_7-8-9_	4.38±1.32*	8.88±1.62	14±1.5	12/12 (100%)
BIOT_PI_	13.67±33.11	6.61±2.69**	29±42.79	10/12 (83%)
BIOT_4PI_	21.5±42.87**	5.78±1.03**	27.1±40.98**	11/12 (92%)

Statistical significance (p < 0.05* and p < 0.01**) compared to control group: CI: control group – infected animals treated with 7% ethanol-water solution diluted in water (10μL/mL) offered *ad libitum*; BIOT_PI_: infected animals treated with biotherapic BIOT_*Tc*17dH_ diluted in water (10μL/mL) offered *ad libitum* from the day of the infection until the death of the animals; BIOT_4PI_: infected animals treated with biotherapic BIOT_*Tc* 17dH_ diluted in water (10μL/mL) offered *ad libitum* from the 4^th^ day of the infection until the death of the animals; BIOT_4-5–6_: infected animals treated with biotherapic BIOT_*TC* 17dH_ by gavage on the 4^th^, 5^th^ and 6^th^ day of the infection; BIOT_7-8–9_: infected animals treated with biotherapic BIOT_*TC* 17dH_ by gavage on the 7^th^, 8^th^ and 9^th^ day of the infection.

Compared to the control group, animals treated with BIOT_PI_ and mainly BIOT_4PI_ showed lower values of area under the curve of daily parasitemia (BIOT_PI_ p < 0.01 and BIOT_4PI_ p < 0.01), lower total parasitemia (BIOT_PI_ p < 0.01 and BIOT_4PI_ p < 0.01), and lower maximum peaks of parasites (BIOT_PI_ p < 0.01 and BIOT_4PI_ p < 0.01) (Table 1). There was no statistical difference in parasitemia on the 8^th^ day of infection and in the 2^nd^ peak of parasites for the BIOT_PI_ and BIOT_4PI_ groups compared to the control, as well as in the percentage of animals that showed the 2^nd^ maximum peak of parasites. These are characteristics typical of infection by the *T. cruzi-Y* strain: a peak of parasites on the 8^th^ day of infection and a second, lower peak of parasites, later. This second peak occurred earlier in BIOT_PI_ and BIOT_4PI_ than in the control group. Considering the parameters shown in Table 2, compared to the control group, the BIOT_PI_ and mainly BIOT_4PI_ groups showed longer prepatent periods (BIOT_4PI_ p < 0.01 and BIOT_PI_ p = 0.13) and shorter patent periods (BIOT_PI_ p < 0.01 and BIOT_4PI_ p < 0.01), longer survival times, significant for the BIOT_4PI_ group (p < 0.01) and a lower (although not significantly lower) mortality. Together, these results show a better outcome of the infection, since the parasitemia in infection by *T. cruzi* is directly proportional to morbidity [17].

In contrast, the BIOT_4-5–6_ and BIOT_7-8–9_ groups showed a worse evolution of the infection compared to the control group. The area under the curve of daily parasitemia in these groups did not differ from the value observed for the control group (p = 0.99), and similarly for total parasitemia (p = 0.99). The BIOT_4-5–6_ and BIOT_7-8–9_ groups showed a higher maximum peak of parasites, significantly higher in the BIOT_4-5–6_ group, as well as a significant advancement of this maximum peak (p < 0.01). There was no significant difference in the parasitemia peak on the 8^th^ day of infection, the parasitemia of the 2^nd^ peak of parasites, nor in the percentage of animals that showed the 2^nd^ peak for the BIOT_4-5–6_ and BIOT_7-8–9_ groups compared to the control group. The prepatent period and survival was lower for these groups compared to the control. Mortality was 100% of the animals, as also observed for the control (Table 2).

Considering the 12 parameters shown in Tables 1 and 2, the BIOT_4PI_ group showed the best overall performance.

In this study, the data obtained for the animals treated with highly diluted medications in the more appropriate treatment schemes (BIOT_PI_ and BIOT_4PI_) showed better results than those observed in the control group. Notably, in the BIOT_PI_ and BIOT_4PI_ groups (which showed better evolution of the infection) there was a significant individual variation observed among the animals of the same group, as shown by the standard deviations (Tables 1 and 2). This individual variation was also observed in the control group.

These data suggest that some animals naturally evolve with lower parasitemia levels, while other animals evolve with higher parasitemia levels. The more-appropriate treatment schemes using highly diluted medications seemed to favor those animals with a tendency to develop lower parasitemia levels. This phenomenon may have led to the result in which the treatment of the BIOT_4PI_ or BIOT_PI_ groups produced a decrease in the parasitemia levels and a tendency to a lower mortality level with a longer survival period. This contrasts with the study by Sandri *et al.* (2010) [18], where a different treatment scheme was used in the same experimental conditions.

### Clinical parameters

The groups of animals that were given the medication (BIOT_*Tc*17dH_) in water *ad libitum* for a long period showed better clinical evolution, with a stable body temperature (p < 0.05), higher weight gain (p < 0.01) and higher water consumption (p < 0.05) compared to the control group. The groups treated for a long period (BIOT_PI_ and BIOT_4PI_) contained more animals with ‘normal-looking’ fur for longer periods during the study, and the BIOT_4PI_ group showed the best results of all. In the BIOT_4-5–6_ and BIOT_7-8–9_ groups, more animals showed bristling hair, with a similar evolution to the control group (CI).

In murine *T. cruzi* infection, the morbidity is linked to parasitemia [17]. However, the host-parasite relationship is essential for the manifestation of the disease, which appears more lethal when the balance of this relationship is jeopardized.

The functional recovery of complex biological systems occurs in an integrated manner (considering the psycho-neuro-immuno-endocrine-metabolic axis), and sometimes the imbalance of one part is necessary to maintain the balance as a whole, with the appearance of new signs and/or exacerbating old symptoms or manifestations [19].

In this context, the host immune response causes many symptoms of the infection [20,21], and may help to explain the discrepancy between virulence and parasite loads [22,23], suggesting new prospects for the advancement of these studies.

### Survival evaluation

Three animals survived in the groups that were given the medication (BIOT_*Tc*17dH_) in water *ad libitum* for a long period. Of these three survivors, two were from the BIOT_PI_ group, and only one of them had patent parasitemia. The other had negative PCR (Polymerase Chain Reaction) [24], understood as a protective action of the drug, since the treatment began at the time of the infection. Considering that the *T. cruzi-Y* strain shows 100% infectivity and mortality in Swiss mice [12,25-27] this result shows a patent benefit of the medication.

The surviving animal in the BIOT_4PI_ group, although it showed a positive PCR diagnosis, never showed patent parasitemia. This result is consistent with low levels of parasitemia and provides information about the benefit of the treatment scheme used, since survival in the murine model is directly related to parasitemia levels [27].

The better evolution of the infection, considering both parasitological and clinical parameters in BIOT_PI_ and mainly in BIOT_4PI_ is expressed by the delayed onset of mortality (16^th^ and 13^th^ day of infection, respectively) as well as the lower mortality compared to the control group. The survival of 3 animals for more than 360 days, with a mean and standard deviation of 27.1 ± 40.98 (p < 0.01) and 29.0 ± 42.79 (p > 0.05) for the BIOT_4PI_ and BIOT_PI_ groups (Table 2), respectively, suggests an improved prognosis in a pathogenesis process that shows an irreversible evolution [28].

### Correlation analysis

The correlation analysis indicates the relationship between two variables within a group. The relationships between each clinical or parasitological parameter (variable) and survival for the groups CI, BIOT_PI_ and BIOT_4PI_ were evaluated separately. This relation is expressed by a number called correlation coefficient (‘r’), which can be significant or not. Their values range from +1 to −1, and its size indicates the force of correlation. Correlations up to 0.299 were considered weak, values from 0.300 – 0.499 were considered intermediate, and strong from 0.500 – 0.999. The signal indicates the direction, if the correlation is positive (direct) or negative (inverse).

Considering that in this model, the evolution of the disease is irreversible, culminating in the death of the animals, an increase in the survival period is an indicator of a good treatment scheme. Some clinical and / or parasitological parameters may be more or less related to this increase in survival. The correlation analysis may indicate which are the more important parameters that could be measured. Therefore, we evaluated the correlations between the parasitological/clinical parameters and survival periods for the control group (CI) and the groups treated with the biotherapic (BIOT_*Tc*17dH_) in the treatment schemes that improved the clinical and parasitological evolution (BIOT_PI_ and BIOT_4PI_) (Table 3).

**Table 3:** **Values of correlation (‘r’) of parasitological and clinical parameters with survival of animals subjected to different treatment schemes using biotherapic (BIOT**_***Tc*17dH**_**)**

**‘r’ FOR CORRELATION**			
**PARAMETERS**	**CI**	**BIOT**_**PI**_	**BIOT**_**4PI**_
AUC	(-)0.2250	(-)0.1980	(-)0.2349
P_total_	(-)0.3220	(-)0.4387	(-)0.5227
P_max_	(-)0.2989	(-)0.3182	(-)0.5350
Day P_max_	NS	NS	NS
PPP	(+)0.4825	(+)0.6704	(+)0.9995
PP	(+)0.7901	(-)0.7000	(-)0.8998
T^a^	NS	NS	NS
WEIGHT	NS	NS	(+)0.4797
WATER	NS	NS	NS

NS = no significant correlation. ‘r’ values are presented for significant correlations (p < 0.05) between survival and the parameters: AUC = area under the curve (parasites/mL); P_total_ – total parasitemia (parasites/ mL); P_max_ = maximum peak of parasites (parasites/ mL); Day of P_max_- Day of maximum peak of parasites; PPP = Prepatent period; PP = patent period; T^a^ = Temperature; Weight and Water. Groups: CI: control group – infected animals treated with 7% ethanol-water solution diluted in water (10μL/mL) offered *ad libitum*; BIOT_PI_: infected animals treated with biotherapic BIOT_*Tc*17dH_ diluted in water (10μL/mL) offered *ad libitum* from the day of the infection until the death of the animals; BIOT4PI: infected animals treated with biotherapic BIOT_*Tc*17dH_ diluted in water (10μL/mL) offered *ad libitum* from the 4^th^ day of the infection until the death of the animals.

The prepatent period (PPP), patent period (PP) and total parasitemia (P_total_) showed moderate to strong correlations with the survival period for all groups. For shorter survival periods, the groups showed higher total parasitemia (P_total_). In groups treated for a long period, the P_total_ was lower and showed increasing ‘r’ values (BIOT_4PI_ > BIOT_PI_ > CI).

The prepatent period (PPP) was best correlated with the survival period. The BIOT_4PI_ group showed a strong and positive correlation (r = 0.9995), indicating that the survival was closely linked to an increase in the prepatent period (PPP). In the different groups, this parameter clearly reflected the differences in performance under the different treatments, showing increasing 'r' values for the BIOT_4PI_, BIOT_PI_ and CI groups. This result, combined with the set of data obtained in this study, indicated that the PPP was the best parameter to evaluate an intervention in this model.

This finding is important considering that in protocols with highly diluted substances, researchers tend to evaluate an excessive number of parameters in an attempt to include the key change that shows a benefit, since benefits may be perceived but cannot always be measured. In the experimental protocol used in this study, the risk of contamination and the workload involved need to be optimized, and it is imperative to select parameters that show the best effects most clearly.

The other parameters evaluated showed weak correlations with survival, although they also showed peculiarities in the comparison among the experimental groups. The maximum peak of parasites (P_max_) was inversely correlated with the survival period; the relationship was moderate for the CI and BIOT_PI_ groups and strong for the BIOT_4PI_ group. These results concord with those for the treatment schemes that began after the fourth day of infection, and again indicate the importance of the treatment scheme in determining the degree of morbidity from the infection.

The correlation between the peak day and the survival period had a particular result: the correlation was strong and direct in the CI group, showing that the later the peak of parasites, the longer is the survival time. In the groups treated with the biotherapic, the correlation was strong, but negative, with a higher ‘r’ value for the group that performed best in this study (BIOT_4PI_ > BIOT_PI_).

This fact is consistent with what we have observed in previous studies: the biotherapic medications seem to anticipate several important biological phenomena in the course of experimental infection with *T. cruzi*[29]. The high standard deviation observed for this parameter in the BIOT_4PI_, BIOT_PI_ and CI groups indicates the individuality of behavior for this parameter (Table 2). Sandri *et al.* (2010) [18] has also observed this behavior individuality in both the treated and control group and found out that the animals that were more resistant to infection had a higher sensitivity to the treatment. In the case of Sandri *et al.* (2010) [18] the treatment scheme was inadequate and resulted in increased parasitemia.

These results show that some parameters can work as ‘markers’ of the performance of an intervention in the infection model used in this study, clearly reflecting the evolution of infection and these parameters can be prioritized in the experimental design, minimizing the work of researchers.

Although in this study in mice, the clinical parameters of temperature, weight and water consumption, as measured, were not effective to clarify the best therapeutic scheme using BIOT_*Tc*17dH_, the researcher perceive the difference among the groups. Find better methods to evaluate these parameters is an important perspective.

Considering the groups of animals treated for long periods, the BIOT_4PI_ group presented a better performance with an intermediate correlation between weight and survival. These data, besides reflecting more accurately what was observed in the clinical evaluations, also supports a good prognosis for animals treated with this scheme.

### Further discussions

The differences observed when comparing the evolution of infection in the groups treated for a long period in water (BIOT_PI_ andBIOT_4PI_), and for a short period of time by gavage (BIOT_4-5–6_ and BIOT_7-8–9_) show a better balance for animals treated for longer periods in water. The discussion of these results can also be based on the fact that the medication administered in water for a long period, besides enabling a higher number of stimuli, also causes less stress to the animal compared to the treatment by gavage, for a short period of time. Also in relation to medication administration, diluted in water, the difference in the intake among the animals, although it may be criticized, According to some authors, the concept of medication dose as the amount of medication that a patient must take to modify his state of illness is not applicable to highly diluted medications, because they do not act based on their mass, but rather by their dynamic effect (qualitative), which continues for a shorter or longer period according to the power of reaction or sensitivity of the organism and the stage of the disease [22,23].

Our data concord with the findings of other investigators that the use of highly diluted medications in the acute phase of infection requires a higher frequency of administered doses [23]. In a randomized controlled experiment, the benefit of the treatment using highly diluted medication continuously and for long periods of time, without interruption of the stimulus, promotes balance in acute infections [23].

Considering that the stimulation provided by the highly diluted medication is related to its ability to affect the psycho-neuro-immune-endocrine-metabolic axis, stress may also be a destabilizing factor that should be considered in evaluating the results [15].

*T. cruzi* is an obligate intracellular parasite and its interaction with the host cells is complex, and inflammation is a decisive component of the pathogenesis. In developing resistance to *T. cruzi,* the immune system can act through different routes that function more or less effectively, depending on the conditions imposed by the host-parasite relationship [17]. Sandri *et al.* (2011) [30] demonstrated modulation of the inflammation process in eight-week-old animals infected with *T. cruzi* and treated with a biotherapic, with a higher rate of apoptosis and reduced production of the cytokine transforming growth factor beta (TGF-β).

In the present study, assuming that the highly diluted medication BIOT_*Tc*17dH_ modulated the host immune system, the finding that the best therapeutic result was achieved with the medication administered beginning after 4 days of infection and for long periods (BIOT_4PI_) is consistent with the following hypothesis: In this scheme, the host's immune system, already activated by the parasite and affected by the highly diluted medication, leads the infection to evolve less aggressively, as described by Sandri *et al.* (2011) [30].

## Conclusion

The BIOT_4DI_ group showed the best clinical and parasitological evolution, with lower parasitemia and a trend toward lower mortality with longer periods of survival. The PPP (prepatent period = time in days from the infection until the first parasite was detected in the blood) proved to be the best parameter to evaluate the effect of a medication in this model.

## Methods

### Animals and infection

The study involved 60, 28-day-old male Swiss mice from the Central Animal Facility of the Universidade Estadual de Maringá. The animals were infected intraperitoneally with 1,400 blood trypomastigotes of *T. cruziY* strain [12].

### Groups according to treatment

The animals were divided equally into five groups according to the treatment: CI - infected animals treated with 7% ethanol-water solution diluted in water (10 μL/mL) offered *ad libitum* in an amber bottle; BIOT_PI_ -infected animals treated with BIOT_*Tc*17dH_ diluted in water (10 μL/mL) offered *ad libitum* in an amber bottle during a period that started on the day of infection and finished when the animals died; BIOT_4DI_ - infected animals treated with BIOT_*Tc*17dH_ diluted in water (10 μL/mL) offered *ad libitum* in an amber bottle from the 4^th^ day of infection to the death of the animals; BIOT_4-5–6_ - infected animals treated with BIOT_*Tc*17dH_ administered by gavage (0.2 mL / animal / day) on the 4^th^, 5^th^ and 6^th^ days after infection; BIOT_7-8–9_ - infected animals treated with BIOT_*Tc*17dH_ administered by gavage (0.2 mL / animal / day) on the 7^th^, 8^th^ and 9^th^ days after infection.

The different treatment schemes were based on the effect of medication linked to the immunological effect [3,18] and on the specific evolution of *T. cruziY* strain in Swiss mice [12,27]. Thus, BIOT_PI_ and BIOT_4PI_ simulated a situation where the medication is offered when the immunological system is not in contact with the parasite, and a situation where the medication is offered when the immunological system is already in contact with the parasite, respectively. In BIOT_4-5–6_ and BIOT_7-8–9_, the medication was offered to animals just prior to the parasite peak and exactly coincident with the parasite peak, respectively [12,27].

### Experimental design

The experiment was performed twice as a blind randomized controlled trial. The animals were divided into separate cages so that the mean initial weights of mice in each group were statistically equal. The animals were kept in a climate-controlled vivarium, under controlled temperature (22.7 ± 1.2 °C), light/dark cycles of 12 hours, and water and food offered *ad libitum*.

### Biotherapic T. cruzi 17 dH (BIOT_Tc17dH_)

The highly diluted medication was produced from the buffy coat of blood collected from the orbital plexus of three mice, containing blood trypomastigotes on the 7th day of infection with *T. cruzi Y-*strain. The BIOT_*Tc*17dH_ was prepared by adding 0.9 mL of *T. cruzi* concentrate (4.1 x 10^7^ trypomastigotes /mL) in 9.1 mL of distilled water in a 30-mL glass bottle. Subsequently, dilutions were made in a 70% ethanol-water solution until the 16dH dynamization. The dynamization used, 17dH (corresponding to a dilution of 1:10^17^ of the initial suspension) was prepared with 7% ethanol-water solution. [8] The microbiological test and the biological risk *in vivo* were negative, according to the regulations of the Brazilian Ministry of Health (RDC No. 67) [31], and the drug was stored in amber glass for better conservation.

### Parameters analyzed

Parasitological and clinical parameters were evaluated. Parasitemia was evaluated by daily counts of blood trypomastigotes from the day of infection, using the technique of Brener (1962) [32]. For animals that showed negative parasitemia, blood was collected for PCR diagnosis (Polymerase Chain Reaction) [24].

Using the parasitemia curve, the following data were obtained: prepatent period (PPP), which is the time from infection to detection of the parasite in blood; patent period (PP), the period when the parasitemia can be detected; and total parasitemia (P_total_), calculated as the sum of the mean daily parasitemia levels for each mouse in each group. The area under the curve (AUC), defined as the number of parasites/ mL of blood observed at a certain time of infection, was calculated using the software Prism Graph 5. The peak of maximum parasitemia (P_max_), considered the mean of the highest level of parasitemia observed in each animal throughout the experiment, parasitemia on the 8^th^ day of infection, the 2^nd^ peak of parasites, and the percentage of occurrence of this 2^nd^ peak of parasites. The parasitemia was expressed in parasites/ mL and the occurrence of this 2^nd^ peak was expressed by the percentage of animals that showed a peak (n) within a group (N). The day on which these peaks occurred was also considered. Mortality was computed during the course of the experiment, resulting in the following parameters: days of survival and percentage of mortality expressed by the number of animals died (n) within a group (N). The animals were clinically evaluated on alternate days from the day of infection until the end of the treatment for body temperature, water consumption, fur appearance, and body weight [33].

### Statistical analysis

The results were compared statistically using the program Statistica 7.0, and the Kruskal-Wallis test (ANOVA followed by multiple comparisons of mean ranks for all groups) was used since the analyzed parameters did not all show a normal distribution. The correlations were analyzed using the R.2.10 program. Correlations with ‘r’ value up to 0.299 were considered weak, from 0.300 – 0.499 as intermediate, and from 0.500 – 0.999 as strong. The acceptable level of significance was 5% for all analyses.

### Ethics

The experiment was approved by the Ethics Committee for Animal Experimentation at the Universidade Estadual de Maringá (UEM), under protocol number 030/2008.

## Abbreviations

BIOTTc17dH, Biotherapic T. cruzi 17 dH; BIOTDI, Group treated with BIOTTc17dH diluted in water offered ad libitum during a period that started on the day of infection and finished when the animals died; BIOT4DI, Group treated with BIOTTc17dH diluted in water offered ad libitum from the 4th day of infection to the death of animals; BIOT4-5–6, Group treated with BIOTTc17dH administered by gavage on 4th, 5th and 6th days after infection; BIOT7-8–9, Group treated with BIOTTc17dH administered by gavage on 7th, 8th and 9th days after infection; CI, Control of infection; AUC, Area under the curve; Ptotal, Total parasitemia; Pmax, Maximum peak of parasites; Day of Pmax, Day of maximum peak of parasites; P8thDI, Parasitemia of 8th day of infection; 2nd Pmax, Second maximum peak of parasites; Day of 2nd Pmax, Day of 2nd maximum peak of parasites; PPP, Pre patent period; PP, Patent period.

## Competing interests

The authors declare that they have no competing interests.

## Authors’ contributions

All authors are key members of the research consortium and have made substantial contributions to the conception and the design of the study, and acquisition of the baseline data. They have all been involved in writing the manuscript and have given their approval for the final version.
